# Mapping the de-implementation of traditional diagnostic tests in pediatric acute lymphoblastic leukemia

**DOI:** 10.3389/fonc.2026.1885970

**Published:** 2026-08-17

**Authors:** Marta Salek, Kenneth Busby, Nathaniel Webb, Dylan E. Graetz, Charles G. Mullighan, Thomas B. Alexander, Nickhill Bhakta, Megan C. Roberts, Lu Wang

**Affiliations:** 1 Department of Oncology, St Jude Children’s Research Hospital, Memphis, TN, United States; 2 Department of Global Pediatric Medicine, St Jude Children’s Research Hospital, Memphis, TN, United States; 3 Department of Pediatrics, Emory University School of Medicine, Atlanta, GA, United States; 4 Department of Pediatrics, University of North Carolina at Chapel Hill, Chapel Hill, NC, United States; 5 Department of Pathology, St Jude Children’s Research Hospital, Memphis, TN, United States; 6 Eshelman School of Pharmacy, University of North Carolina at Chapel Hill, Chapel Hill, NC, United States

**Keywords:** cancer diagnostics, clinical genomics, cytogenetics, de-implementation, diagnostic optimization, pediatric cancer, process mapping

## Abstract

**Introduction:**

Advances in cancer diagnostics raise questions about when and how to de-implement traditional approaches; however, these processes remain poorly described. At St. Jude Children’s Research Hospital (SJCRH), routine conventional cytogenetics for pediatric acute lymphoblastic leukemia (ALL) diagnosis was de-implemented in 2018 following adoption of clinical genomics. This study aimed to map this process to inform future diagnostic de-implementation initiatives.

**Methods:**

Interviews were conducted with SJCRH staff involved or impacted by cytogenetics de-implementation. Data were analyzed using thematic and rapid qualitative analysis informed by the Consolidated Framework for Implementation Research. Member-checking was used to verify and refine process maps, which were subsequently reviewed by an external expert panel, representing diverse settings, through focus group discussions.

**Results:**

Thirteen SJCRH clinicians participated. De-implementation was described as successful, with no negative impact on patient outcomes. Decision-making began with internal correlation studies that demonstrated superior diagnostic performance of clinical genomics. De-implementation was viewed as a natural evolution that improved molecular classification, resource allocation, and workflow efficiency. Perceived risks included loss of cytogenetics competency, delayed turnaround time, and career insecurity, all addressed institutionally. Lessons learned highlighted the importance of deliberate discussion about logic and evidence supporting de-implementation. Fifteen external experts offered suggestions to improve process map generalizability, highlighting institutional- and system-level considerations.

**Conclusion:**

De-implementation of cytogenetics in ALL in favor of clinical genomics was successful at SJCRH. This study offers an example of diagnostic de-implementation in cancer care and proposes a structured approach to guide future efforts. De-implementation should be considered alongside introduction of novel diagnostic approaches.

## Introduction

The diagnosis and classification of pediatric cancer has traditionally relied on multiple diagnostic tests performed concurrently, each providing discrete information that together supports cancer identification (1). Each test requires distinct equipment, specialized technical and interpretive expertise, and incurs additive capital and consumable costs (1). Modern next-generation sequencing (NGS) assays challenge this paradigm (2–6). Although these assays generate novel diagnostic information at the individual-sample level, the resulting data, when analyzed using different computational pipelines, can yield findings equivalent to those obtained with conventional techniques such as karyotyping, fluorescence *in situ* hybridization (FISH), and reverse transcription polymerase chain reaction (RT-PCR), creating duplication and redundancy in diagnostic reporting (7, 8). Eliminating such redundancies could improve resource allocation, reduce costs, and accelerate cancer classification and treatment planning (1).

At St. Jude Children’s Research Hospital (SJCRH), real-time paired tumor-normal clinical genomics testing was introduced in 2017 and offered to all pediatric cancer patients receiving care at the institution or enrolled on SJCRH-sponsored clinical trial. This platform integrates of whole-genome (WGS), whole-exome (WES), and whole-transcriptome sequencing (WTS or RNA-seq). When compared with conventional cytogenetics, WGS demonstrated higher sensitivity for detecting chromosome translocations and higher resolution for copy-number analysis, while also resolving complex derivative chromosomes observed in karyotyping. Whole-transcriptome sequencing also demonstrated clinically acceptable turnaround times relative to FISH and RT-PCR, decreasing from approximately two weeks initially to four-to-six-day turnaround for key acute lymphoblastic leukemia (ALL) driver fusions. Due to the superiority of this integrated platform, clinical genomics results informed clinical management and a decision was made institutionally to discontinue conventional cytogenetics for the diagnosis of pediatric ALL at SJCRH in 2018. Collectively, WGS replaced conventional karyotyping and FISH probes for detecting structural rearrangements, copy-number alterations, and aneuploidies; WTS replaced RT-PCR and complemented WGS for fusion detection while also providing gene expression–based subtype classification not achievable by conventional methods; and WES performed alongside WGS and WTS enhanced the analytical sensitivity of detecting exonic variants at low allele fraction. This consolidated diagnostic workflow enabled comprehensive testing from a single specimen, reducing testing redundancy, improving turnaround time, and enhancing disease classification and risk stratification.

De-implementation science provides a framework for removing, replacing, reducing, or restricting an ineffective or potentially harmful practice and is distinct from implementation science, which focuses on introducing an evidence-based intervention into clinical settings (9, 10). Despite its relevance, de-implementation of routine interventions in pediatric cancer and its impact on care delivery remains largely unexplored, and practical frameworks to guide de-implementation are limited (11, 12). The discontinuation of conventional cytogenetics at SJCRH offers an opportunity to retrospectively map decision-making and actions taken during de-implementation, particularly given that cytogenetics remains a core component of pediatric ALL globally.

In this qualitative study, a two-phase approach was used to: 1) map the process of de-implementing conventional cytogenetics in the diagnosis of pediatric ALL at SJCRH and 2) develop a framework to guide future efforts to de-implement selected interventions in cancer, incorporating feedback from international childhood cancer experts. Feedback from external experts was solicited to enhance the generalizability and applicability of the proposed framework for use across diverse centers and health systems.

## Methods

### Study design

This study was reviewed and approved as exempt by the Institutional Review Board at SJCRH, given the use of de-identified data collection with adult participants. The study protocol was designed in two phases by a research team comprising of experts in pediatric oncology, pathology, global health, and de-implementation.

Process mapping visually represents the relationship among activities, people, and resources and identifies bottlenecks from diverse perspectives and stakeholders (13). Process maps were developed following best practices and stages identified in a systematic review, including preparation, planning, data collection, map generation, analysis, and dissemination (14). To comprehensively understand the multilevel and multiphase nature of diagnostic de-implementation, study design was informed by three complementary frameworks and models: (1) the Consolidated Framework for Implementation Research (CFIR), a determinant framework that identifies multilevel barriers and facilitators to change; (2) the Exploration, Preparation, Implementation, and Sustainment (EPIS) framework, a process model used to organize process steps, determinants, and behavioral targets into distinct phases; and (3) the Integrated Behavioral Model, which supports identification of individual-level behavioral mechanisms relevant to de-implementation (15–20).

In Phase 1, the de-implementation of conventional cytogenetics in pediatric ALL at SJCRH was examined. Semi-structured interviews with eligible SJCRH staff informed the creation of two process maps: (1) depicting the de-implementation of conventional cytogenetics in pediatric ALL at SJCRH and (2) outlining a proposed framework for de-implementing future diagnostic interventions in cancer. Interview participants were subsequently invited to a member-checking focus group to validate and refine the process map of the cytogenetics de-implementation experience and to provide feedback on the proposed framework (21).

In Phase 2, an international panel of external experts in pediatric oncology and pathology participated in focus groups to review and provide feedback on both process maps developed in Phase 1. This review sought to incorporate expert perspectives on the SJCRH experience and to refine the proposed framework so it could be adapted beyond a single, well-resourced center and applied across diverse healthcare settings.

### Setting and participants

In Phase 1, a purposive sample of SJCRH staff was recruited from laboratory operations and the Departments of Oncology and Pathology. SJCRH is a pediatric treatment and research institution dedicated to the care of children diagnosed with cancer and other life-threatening illnesses. On average, SJCRH cares for 600 newly diagnosed pediatric cancer patients annually, including approximately 70 children with ALL. Eligible participants were individuals involved in or impacted by the de-implementation of cytogenetic testing in pediatric ALL at SJCRH, including current or former employees. Those who declined participation due to ineligibility were asked to recommend other eligible individuals. Recruitment was conducted iteratively; interviewed participants were asked to identify additional individuals who had played important roles in or were affected by the de-implementation process.

In Phase 2, participants with expertise in pediatric ALL, childhood cancer, or pathology were identified from the St. Jude Global Alliance community, an international network of >300 medical institutions and foundations from >80 countries, dedicated to improving outcomes for children with catastrophic illness (22). Additionally, a purposive sample of eligible experts outside of the Alliance was recruited to ensure representation across diverse world regions and country income levels (23).

During both phases, data collection and analysis occurred concurrently to assess whether new process steps, determinants, or perspectives continued to emerge. Recruitment continued until thematic saturation was reached, defined as the point at which no substantively new themes or insights were identified (24, 25). Eligible participants were contacted by a member of the research team (M.S.) via e-mail, and informed consent was obtained from each participant prior to initiating qualitative data collection.

### Interview and focus guide development

The semi-structured interview guide used in Phase 1 included questions and probes aligned with key constructs from the updated CFIR (**Supplementary Table 1**) (15, 16). Constructs within the Implementation Process domain were emphasized in order to develop an initial process map, along with broader questions to identify barriers and facilitators across CFIR domains: inner setting (e.g., hospital environment), outer setting (e.g., policies), innovation characteristics (e.g., cost), and individual-level factors (e.g., motivation) (15, 16). To enhance assessment of individual-level determinants influencing de-implementation behaviors, the CFIR individual characteristics domain was supplemented with constructs from the Integrated Behavioral Model, including personal agency, attitudes, and norms (20). Member-checking focused on soliciting feedback on the accuracy, clarity, and comprehensibility of the developed process maps (**Supplementary Table 2**).

The Phase 2 focus group guide was informed by the adapted CFIR incorporating constructs relevant to low- and middle-income country settings (**Supplementary Table 3**) (19). Participants were asked to comment on the clarity and comprehensiveness of the process maps, share impressions on the de-implementation process and its contextual fit, and comment on the acceptability and appropriateness of discontinuing routine cytogenetics for pediatric ALL at their centers.

### Data collection

All interviews and focus groups were conducted in English via a secure, online video-conferencing platform and were audio-recorded and transcribed. Sociodemographic data were collected in Phase 1 by the research team (M.S., K.B.) during interviews and included gender, age, years of clinical practice, specialty, and institutional role. In Phase 2, additional demographic variables were obtained via electronic survey, including country of practice, hospital funding source, annual pediatric cancer and ALL case volume, and routine use of cytogenetics for pediatric ALL diagnoses.

Phase 1 interviews were conducted by trained experts in qualitative data collection (M.S., K.B.), without supervisory or hierarchical relationships to participants, between April and June 2024 and lasted 30–60 minutes. Member-checking sessions were held in September 2024. Phase 2 focus groups (facilitated by M.S., M.R.) were conducted in September and October 2024, with four sessions organized to accommodate time zones and availability. Each session lasted 20–60 minutes.

### Data analysis

Demographic characteristics were summarized descriptively. Phase 1 interviews were de-identified prior to analysis. A rapid qualitative analysis approach was used to summarize and interpret transcript data, a method widely used in implementation science to enable timely synthesis of interviews to inform process mapping for expert review (26). Summary templates were organized by interview guide question and synthesized into a matrix by participant and CFIR construct, which informed the bases of the process maps (**Supplementary Table 4**). Tabulation techniques were used to identify patterns, variations, and themes. Findings were organized into four stages informed by the EPIS framework: exploration, planning, de-implementation, and sustainability (17, 18).

Process maps were created using a virtual whiteboard platform by two research team members (M.S., M.R.) (27). De-implementation determinants were incorporated as annotated facilitators and lessons learned. Swim lanes were used to organize de-implementation steps by responsible department. A second, more generalizable process map was iteratively developed to guide future diagnostic de-implementation efforts. Both maps were reviewed during member-checking sessions and Phase 2 focus groups, with feedback incorporated into final revisions.

Phase 2 focus group transcripts were independently reviewed by two researchers (M.S., M.R.), who conducted memo writing and thematic analysis to identify high-level themes related to diagnostic de-implementation and the proposed process maps (28).

## Results

### Internal process map of cytogenetics de-implementation in pediatric ALL at SJCRH

**Figure 1:**
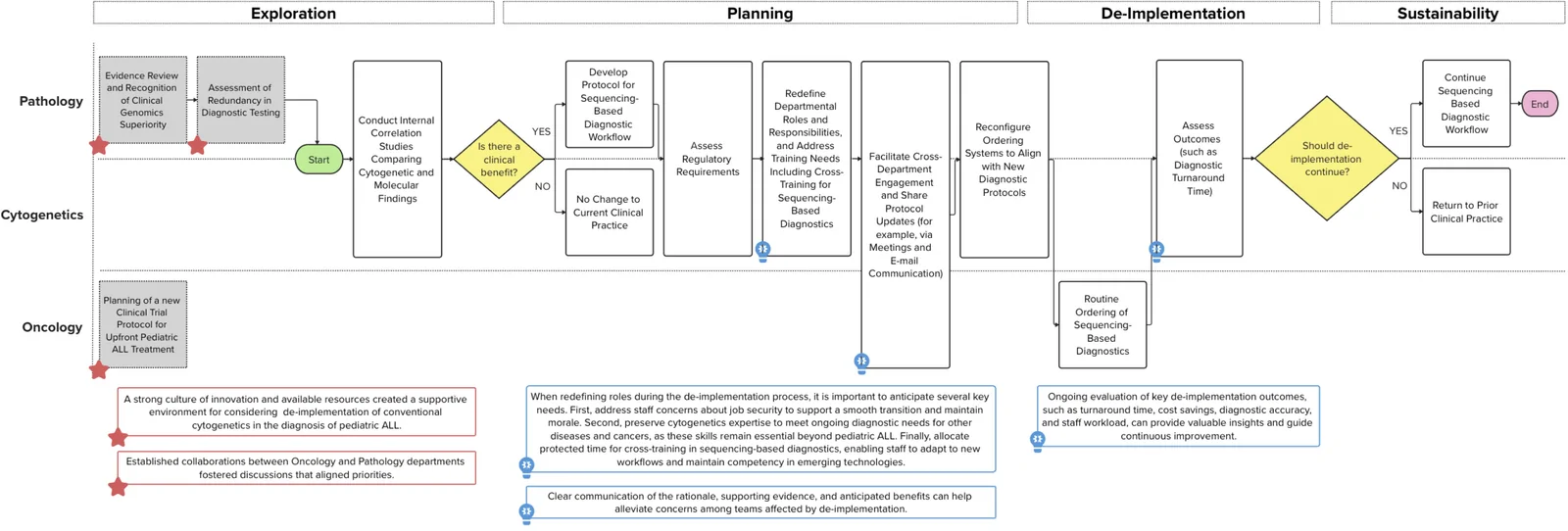
Process map of conventional cytogenetics de-implementation in pediatric acute lymphoblastic leukemia at St. Jude Children’s Research Hospital (SJCRH). The process map is organized into four de-implementation stages: exploration, planning, de-implementation, and sustainability. Swim lanes are used to organize de-implementation steps by responsible department.

Thirteen current or former SJCRH employees participated in interviews; participant demographic information is summarized in **Table 1**. Participants described de-implementation of conventional cytogenetics in pediatric ALL as involving the Departments of Oncology and Pathology, specifically the Clinical Cytogenetics Laboratory. These insights informed the structure of the process map, including the delineation of swim lanes corresponding to discrete process steps taken by different departments. Process map components distinguished activities occurring prior to de-implementation from those undertaken during de-implementation and incorporated participant reflections on lessons learned and process facilitators (**Table 2**). Activities were organized into four stages: exploration, planning, de-implementation, and sustainability (**Figure 1**). During member-checking, feedback indicated that the process map was easy to follow, clearly presented, and accurately reflected the de-implementation experience at SJCRH.

**Table 1:** Participant demographic for Phase 1 (current and former employees of St. Jude) and 2 (international experts).

Characteristic		n (%)
Phase 1 – process mapping with current or former SJCRH employees		
Age*	36-50	3 (23)
	51-64	3 (23)
	≥65	6 (46)
Gender	Female	5 (38)
	Male	8 (62)
Area of Specialty	Oncology	6 (46)
	Pathology	7 (54)
Years Practiced in Specialty	6-10	1 (8)
	11-15	2 (15)
	16-20	2 (15)
	≥21	8 (62)
Holds Leadership Position	Yes	12 (92)
	No	1 (8)
Currently SJCRH Employee	Yes	10 (77)
	No	3 (23)
Phase 2 – process map review with international expert panel^#^		
Individual-level characteristics		
Age*^#^*	21-35	1 (7)
	36-50	4 (29)
	51-64	8 (57)
Gender*^#^*	Female	9 (64)
	Male	4 (29)
Area of Specialty	Hematology/Oncology	6 (40)
	Pathology	9 (60)
Years Practiced in Specialty*^#^*	0-5	1 (7)
	6-10	1 (7)
	11-15	2 (14)
	16-20	3 (22)
	≥21	6 (43)
Holds Leadership Position*^#^*	Yes	8 (57)
	No	5 (36)
Setting-level characteristics		
Country Income Level	LMIC	4 (27)
	UMIC	4 (27)
	HIC	7 (46)
World Health Organization Region	Americas	4 (27)
	Eastern Mediterranean	1 (7)
	Euro	6 (39)
	Sub-Saharan Africa	1 (7)
	Western Pacific	3 (20)
Institution Funding Source*^#^*	Private	1 (7)
	Public	7 (50)
	Both	5 (36)
Annual Number of New Childhood Cancer Cases*^#^*	21-49	1 (7)
	50-99	1 (7)
	100-299	3 (22)
	≥300	6 (43)
	Unsure	2 (14)
Annual Number of New Pediatric ALL Cases*^#^*	≤20	2 (14)
	21-49	2 (14)
	50-99	2 (14)
	100-299	3 (22)
	≥300	1 (7)
	Unsure	2 (14)
Cytogenetics Routine Part of ALL Work-Up Locally*^#^*	Yes	8 (57)
	No	5 (36)

*Of 13 Phase 1 participants, one declined to share their age.

^#^ Of 15 Phase 2 participants, two participants did not complete the demographic survey.

SJCRH, St. Jude Children’s Research Hospital; LMIC, lower-middle-income country; UMIC, upper-middle-income country; HIC, high-income country; ALL, acute lymphoblastic leukemia.

**Table 2:** Symbols used in developing the de-implementation process maps.

Symbol	Name	Function
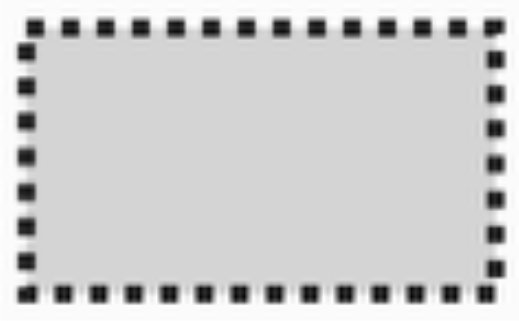	Process	A dotted gray rectangle represents a subprocess that occurs before de-implementation begins.
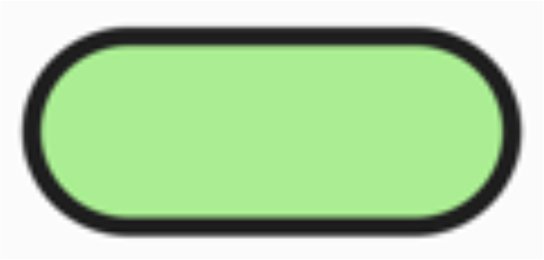	Start	A green oval represents the start point of the de-implementation process.
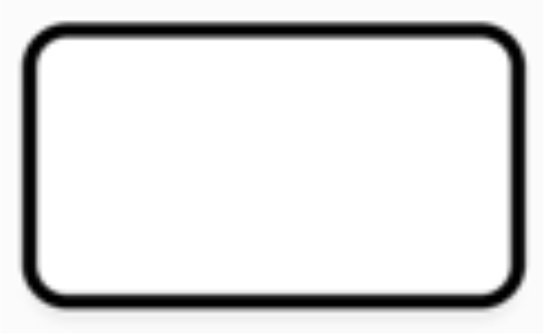	Process	A white rectangle represents a subprocess that occurs during the de-implementation process.
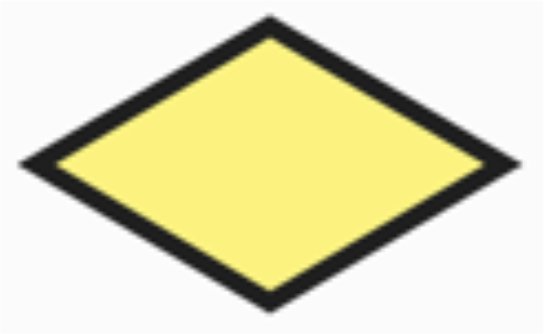	Decision	A yellow diamond represents a decision during the de-implementation process.
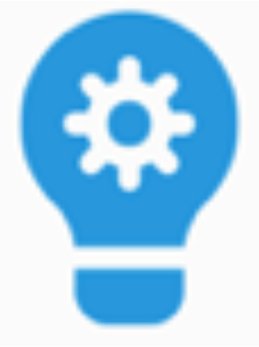	Lesson Learned	A blue light bulb represents a lesson learned during the de-implementation process.
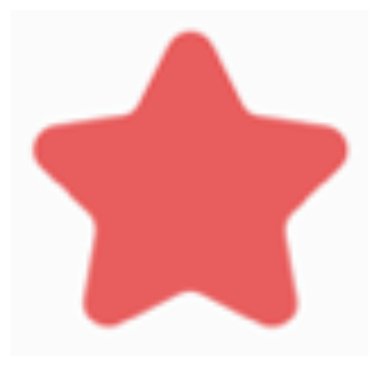	Facilitator	A red star represents a condition or determinant that enables de-implementation to occur.
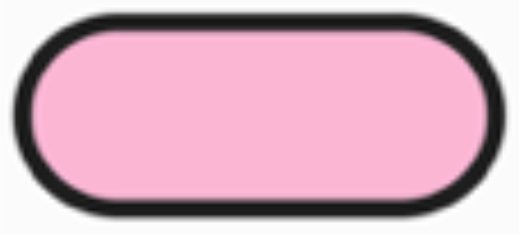	End	A pink oval represents the end point of the de-implementation process.

### Exploration

During exploration, two concurrent initiatives were identified within the Departments of Oncology and Pathology. The Department of Pathology had implemented RNA-Seq and WGS alongside cytogenetic testing for the diagnosis of ALL. Internal correlation studies demonstrated that clinical genomics provided superior diagnostic information compared with cytogenetics, with acceptable turnaround times. These findings revealed redundancy between diagnostic approaches, prompting discussion of de-implementation: “we began to discuss the potential for winding down conventional cytogenetics in this setting given that it provided far less information than NGS” (Phase 1, Participant 1). This led to consideration of whether cytogenetics could be safely de-implemented and replaced by clinical genomics in the upfront diagnostic work-up of pediatric ALL. At the same time, the Department of Oncology was developing a new clinical trial protocol for the upfront diagnosis and evaluation of pediatric ALL, creating an additional opportunity to reassess diagnostic strategies.

Key facilitators included a culture of innovation, adequate resources and infrastructure, and strong interdepartmental collaboration, which supported alignment of departmental priorities. These priorities included delivering optimal patient care, improving “sample utilization [and diagnostic] workflows in terms of efficiency and cost, [ … preserving] genomic accuracy and avoiding waste” (Phase 1, Participant 12). Consideration of cytogenetic de-implementation in response to emerging technologies was viewed as a natural evolution of diagnostic practice.

### Planning

Planning began when the Department of Pathology questioned whether clinical genomics provided added clinical benefit over conventional cytogenetics at diagnosis of ALL. In response, the Department of Pathology developed a sequencing-based diagnostic protocol and evaluated relevant regulatory requirements. Planning activities required reallocation and clarification of roles and responsibilities across both departments, including preparation for increased testing volume and associated workforce training needs.

Key workforce determinants included concerns about job security during a major shift in diagnostic workflows (with no staff positions ultimately eliminated) and the need to preserve cytogenetics expertise. These concerns were evident at both individual- and departmental-levels. At the individual-level, staff reported uncertainty about their roles: “[staff within the Cytogenetics department] started feeling that [it] was not a safe job [ … they] felt very uncertain of their futures” (Phase 1, Participant 13). From a leadership perspective, shifts in workload and how best to manage specialized personnel were key considerations, with one participant noting, “[in my department], I had people [with] specialized expertise in this area who were going to see a big change in their workload. One had to be mindful about how to manage that” (Phase 1, Participant 1).

While conventional cytogenetics was replaced by clinical genomics for ALL, it was recognized that cytogenetics would remain necessary for selected indications (e.g., early-relapse cases with relatively low blast counts) or for other pediatric cancers. The importance of protected time to support staff training, certification in conducting molecular testing, workflow adaptation, and maintenance of competency in cytogenetics was noted. Planning also included cross-departmental engagement and communication via meetings and e-mail to coordinate process changes. Oncology participants described uncertainty regarding interpretation of genomics findings and the need for clinician-friendly reports that were understandable and supported clinical decision-making.

Lessons learned underscored the importance of early, transparent communication about the rationale, supporting evidence, and anticipated benefits of de-implementation to proactively mitigate concerns, build trust, and support buy-in, particularly for practices historically central to ALL diagnosis. For example, one participant highlighted the need for early involvement: “I would have liked to be involved in some of the [planning] conversations … [to] have been knowledgeable about what was going on and [mitigate] trust issues between [team members]” (Phase 1, Participant 3). Others described initial skepticism: “I was skeptical that NGS could completely match conventional methodology in terms of [ALL] classification (Phase 1, Participant 11). Conversely, confidence increased with collaborative validation of genomic findings, with one participant stating: “I was very happy to see many people [and departments were] involved with [ … ] implementation [of clinical genomics] and [had] confirmed [genomic findings with cytogenetics results]. That increased my confidence [understanding] the process of how it was done and how many people were involved” (Phase 1, Participant 11). Finally, participants emphasized the need for stepwise, inclusive communication, with one noting: “once a decision [has been made], bring in the people [ … ] little by little, start communicating, say what is going to happen, how it’s going to happen [ … ] bring the people in so [they] can buy-in to the process. I think [it] will be easier for everybody” (Phase 1, Participant 13).

This stage concluded with reconfiguration of ordering and reporting systems to align with the proposed de-implementation.

### De-implementation

De-implementation was initiated with the launch of clinical genomics as the standard approach for upfront diagnosis of pediatric ALL and routine clinician ordering of sequencing-based diagnostics. Diagnostic accuracy was evaluated by correlating clinical genomics results with conventional cytogenetics, which supported adoption of clinical genomics as the primary diagnostic modality. Cytogenetic processing was retained as a contingency strategy through stored sample pellets, prepared for analysis when sequencing results were non-diagnostic or required confirmation; however, these pellets were ultimately not used in clinical practice. No adverse impact on patient care or clinical outcomes was identified during this transition.

During early de-implementation, cross-departmental communication was supported through biweekly meetings held over approximately one year, during which Pathology and Leukemia clinicians reviewed 5–10 cases per session. These meetings facilitated shared interpretation of clinical genomics results and supported clinical application of findings. Conducting cytogenetics in parallel with NGS during the initial transition period increased clinician confidence in the new diagnostic approach, as one participant noted “there were a lot of questions that were asked to make sure we were getting the data we [needed]. We were all cautious, [ … ] the parallel testing was enough to persuade people [the change] was likely to be effective. We learned a lot [from] our pathology colleagues to give us that reassurance, and our trust in them and the relationships we had enabled us to [feel] comfortable” (Phase 1, Participant 7). Challenges related to communicating clinical genomics results to patients and families were also recognized and became the focus of a separate research initiative. Oncology clinicians further reported that direct, accessible communication with Pathology colleagues enabled timely, case-based problem-solving, a practice that may be difficult to replicate in other institutional contexts.

Additional implementation outcomes assessed during this stage included turnaround time, case volume, cost implications, and staff workload. Proactive monitoring of these metrics in future initiatives could provide critical feedback on process performance and support ongoing monitoring and optimization.

### Sustainability

The transition from de-implementation to sustainability was marked by consideration of whether discontinuation of conventional cytogenetics in pediatric ALL could be maintained over time. Sustainability was described as including ongoing monitoring of key outcomes (e.g., turnaround time) to support continued refinement of diagnostic workflows and adaptation to evolving team and system needs.

### Process mapping of future de-implementation of a chosen diagnostic intervention

**Figure 2:**
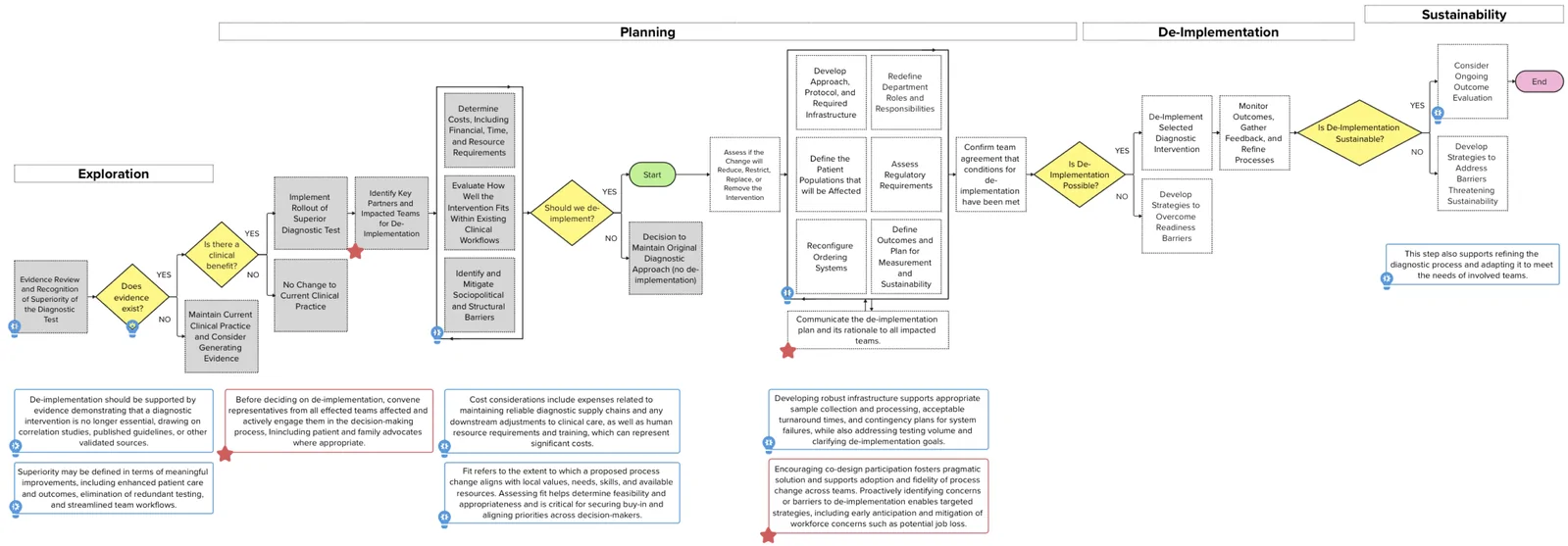
Proposed process for de-implementation of a diagnostic intervention. The process map is organized into four de-implementation stages: exploration, planning, de-implementation, and sustainability. Swim lanes are not included, as these are context-specific and intended to be defined locally.

Drawing on the de-implementation experience at SJCRH, a framework was developed to guide future teams in de-implementing diagnostic tests (**Figure 2**). The process framework reflects the same four stages identified previously but does not include swim lanes, as these were considered context-specific and intended to be defined locally.

In the exploration stage, teams assess available evidence to determine the relative clinical value of a diagnostic test. De-implementation decisions should be supported by evidence, including internal correlation studies, published guidelines, or other validated sources. Clinical superiority may be defined by meaningful improvements such as enhanced patient care and outcomes, elimination of redundant testing, and improved workflow efficiency.

The planning stage involves formal initiation of de-implementation efforts, including identification of key partners and teams affected by the proposed change. Convening a multidisciplinary leadership group, potentially including individuals beyond immediate clinical teams (e.g., patient and family advocates, when appropriate), supports engagement and shared decision-making. Planning activities are focused on identifying salient determinants (e.g., time, financial, or resource costs), assessing fit within existing workflows, and anticipating sociopolitical or structural barriers. Consideration of workforce capacity, training needs, and supply chain reliability is critical. Assessing fit with local values, skills, and resources supports judgments about feasibility and appropriateness and facilitates alignment and buy-in.

The leadership group then determines whether de-implementation is warranted. If so, subsequent planning specifies whether the diagnostic test should be reduced, restricted, replaced, or removed within the local setting. Planning activities focus on operationalization of the change, including defining the de-implementation approach, clarifying roles and responsibilities, identifying the impacted patient population, assessing regulatory requirements, reconfiguring ordering systems, and specifying implementation and sustainability outcomes with corresponding measurement plans. Infrastructure development is critical to de-implementation success, including anticipation of changes to sample collection and processing, turnaround times, contingency planning for system failures, and shifts in testing volume. Clear communication of de-implementation goals and strategies can support alignment across teams. Planning should also include a communication strategy to disseminate the rationale and planned changes to affected groups beyond the leadership group.

This process continues until leadership determines that conditions necessary for de-implementation have been met. Establishing a co-design and engagement-oriented environment can support adoption and fidelity, enable real-time identification of barriers and concerns, and address workforce apprehensions.

Once the leadership team determines that de-implementation is feasible, de-implementation is initiated with ongoing monitoring of outcomes, incorporation of feedback, and iterative process refinement. This stage progresses to consideration of sustainability, including continued outcome evaluation and development of strategies to address barriers that threaten long-term maintenance of the change.

### External review of process mapping

Of twenty-six international experts that were invited to participate in Phase 2, fifteen participated in focus groups. All but two participants completed the requested demographic information. Phase 2 participant characteristics are summarized in **Table 1**.

Overall, Phase 2 experts expressed positive perceptions of both process maps. The process maps were described as easy to follow, clearly presented, and providing pragmatic guidance for approaching de-implementation. Experts viewed the process maps as applicable across settings and potentially adaptable for use in resource-constrained contexts, where optimization of diagnostic processes is critical to maximizing patient benefit.

Experts reflected on de-implementation efforts in their local settings. Some reported difficulty identifying opportunities for de-implementation due to limited resources. Others cited prior experiences, including discontinuation of galactomannan testing for the diagnosis of *Aspergillus* infection. Phase 2 expert impressions of the process maps and perceptions of de-implementation are summarized in **Supplementary Table 5**.

Participating experts additionally identified several considerations for future de-implementation initiatives at both the institutional- and health system-levels (**Table 3**). At the institutional-level, cost was viewed as a key determinant, though perspectives varied. Some emphasized the need to assess cost-benefit early in the planning stage, while others highlighted perceptions that sequencing-based diagnostics were more costly than cytogenetics. In contrast, other participants noted potential cost advantages of newer technologies when accounting for materials, staff time, and turnaround time. Workforce implications were also raised, including concerns about anticipated job losses. Participants further emphasized the importance of institutional readiness, including development of contingency plans to ensure access to external diagnostic testing if services were discontinued locally.

**Table 3:** Considerations identified by external experts for future cancer diagnostic de-implementation initiatives.

Consideration	Quotation
Institution-level	
Cost	If we were to do something like this in my setting, one thing we would consider would be cost [ … ] from the planning phase [ … ] is it worth, spending on this if I’ll get [these] results from [an existing] test. (Phase 2 Participant 1, LMIC)
	In [my country], it is very expensive to shift to NGS-based diagnosis and de-implement cytogenetics, it is much cheaper [ … ] our hospital is based solely on donations and insurance does not cover these kinds of laboratory testing. (Phase 2 Participant 6, LMIC)
	You have to [consider] all the costs, but not only the materials you use, but also the time of people, because some of the newer techniques take much less time than the classic techniques in which a lot of handwork is necessary. (Phase 2 Participant 11, HIC)
Staffing Adjustments	The part about losing jobs would be very important. In our system, we wouldn’t have the capacity to retrain or put these people in other places. So that would be a huge obstacle if we wanted to de-implement something. (Phase 2 Participant 12, UMIC)
	If we say, we’re going to [discontinue] cytogenetic analysis [ … ] instead, we’re going to convert it to completely molecular-based or NGS-method. I think, probably the workforce of senior technologists will lose their jobs here. (Phase 2 Participant 7, HIC)
Institutional readiness	[Let’s] say there is a suggestion [to de-implement in a] guideline, people are going to take this very seriously. Suddenly [they will] stop providing cytogenetic services [ … are] the institutions ready for that? Or do they need to outsource specimens they want to test [ … it is an important] discussion [ … and to] know the market and need. (Phase 2 Participant 7, HIC)
System-level	
Impact on Cancer Guidelines	If we de-implement the conventional strategy, that means no karyotype information will be available [ … that] will jeopardize or interrupt guidelines, especially for clinicians. The [most often] used guidelines, for instance NCCN guidelines, they use [that] information, especially additional chromosomal abnormalities, to characterize risk. (Phase 2 Participant 7, HIC)
	Related to the classification guidelines like [the] WHO and the ICC guidelines for B-ALL. Those are not 100% consistent, there are still some differences [ … are they] prepared for this either transition implementation and de-implementation? (Phase 2 Participant 7, HIC)
Coordination across existing networks	It sounds great, but I think it is [country] dependent. I think it is possible, but it needs well organized cooperation between diagnostic laboratories and clinicians [ … in my country] we have [many] centers. We cooperate very closely to standardize all diagnostic methods. (Phase 2 Participant 5, HIC)
Impact on referral systems	In our hospital, we also receive cytogenetic testing from other laboratories and other hospitals, in different locations in [my country], the flow of cases is like five times our in-patients. It [would] need coordination with other laboratories if we de-implement our cytogenetic testing. (Phase 2 Participant 7, LMIC)

At the system-level, participants highlighted the broader implications of diagnostic de-implementation for treatment guidelines, risk stratification, and cancer care delivery. De-implementation of a foundational diagnostic test such as cytogenetics was viewed as having implications for how existing clinical guidelines are applied. In some settings, alignment across cancer centers within a given country was described as essential, with de-implementation decisions requiring coordination and consensus at regional or national levels. Participants also noted that de-implementation at referral centers could have downstream effects on partner institutions that rely on those centers for diagnostic testing, underscoring the need to consider system-wide impacts. Considerations identified by external experts for future cancer diagnostic de-implementation initiatives are summarized in **Table 3**.

## Discussion

This qualitative study informed the development of two process maps: one describing the de-implementation of a historically standard diagnostic test in pediatric ALL at a single, well-resourced childhood cancer center and a second outlining a potential approach to future diagnostic de-implementation efforts (**Figures 1** and **2**). Feedback solicited from both internal (SJCRH) and external (international) experts found the process maps pragmatic, easy to understand, and broadly applicable (**Supplementary Table 5**). External experts further identified institutional- and system-level considerations relevant to diagnostic de-implementation, including cost and implications for treatment guidelines and care coordination (**Table 3**). These process maps demonstrate that de-implementation can be structured, intentional, and evidence-driven rather than passive, and that sharing institutional experiences may support broader improvements in care delivery.

Advances in cancer diagnostics, including the continued discovery of cytogenetically cryptic driver genetic alterations and molecularly defined gene expression subtypes, present opportunities to reassess the appropriateness and value of established testing practices (29). At SJCRH, de-implementation of conventional cytogenetics in pediatric ALL was safe, with no adverse patient outcomes and comparable or improved turnaround times. Similar advantages of clinical genomics over traditional diagnostic approaches have been reported at other centers (8). As of 2024, routine RT-PCR and FISH assays have also been discontinued in the upfront diagnostic evaluation of pediatric ALL at SJCRH. Moreover, ongoing internal evaluation has demonstrated that WES may be unnecessary when both WGS and WTS are performed on diagnostic samples with high blast counts, although WES may offer enhanced analytical sensitivity for detecting low allele fraction variants (<10%) due to its greater sequencing depth. As genomic technologies continue to evolve, ongoing reassessment of diagnostic practices, and the de-implementation of redundant approaches, will be essential to optimizing patient care, resource utilization, and diagnostic efficiency.

Considering diagnostic de-implementation across diverse resource settings may challenge existing paradigms and inform future pediatric cancer diagnostic approaches. In well-resourced settings, this may involve paired implementation of clinical genomics with de-implementation of traditional diagnostic approaches. In settings without established diagnostic capacity, this may instead involve bypassing cytogenetics altogether in favor of direct adoption of sequencing-based technologies, that are becoming less cost prohibitive and may offer greater scalability and efficiency (30, 31). Implementation of novel technologies also creates opportunities and responsibilities to evaluate de-implementation outcomes, including exploring behavioral determinants that facilitate or hinder the abandonment of traditional diagnostic practices (e.g., openness to change, resistance) (32, 33).

Within the U.S. healthcare system, there is growing recognition of the importance of de-implementation, given estimates that billions of dollars are spent annually on low-value care, including in pediatrics (34–37). Identifying low-value interventions (e.g., that provides little or no benefit, causes harm, incur unnecessary costs, or waste resources), creates opportunities to redirect resources toward higher-value care through de-implementation (38). In oncology, the evidence base for de-implementation remains limited (11, 39, 40). A recent systematic review examining interventions to de-implement low-value oncology care identified few studies, with mixed effectiveness and no representation of pediatric populations (11). Although professional societies have raised awareness through initiatives such as the *Choosing Wisely* campaign, these efforts have been limited by their largely passive strategies and persistent implementation barriers (41, 42). In pediatric oncology, treatment de-escalation trials represent a related effort, demonstrating that less intensive therapy can be safe and effective (43–45). However, these trials do not address how reduced-intensity or discontinued practices are adopted and sustained in routine clinical care (43–45). Prospective application of the proposed process offers an opportunity to address this gap by evaluating de-implementation strategies and examining behavioral and system-level determinants of adoption and sustainment.

These findings should be interpreted in the context of several limitations. In Phase 1, interviews were conducted with SJCRH employees more than five years after initiation of the conventional cytogenetics de-implementation process, introducing potential recall bias.

Archival records were not available to inform study design or process map development, as the de-implementation process had not been formally documented or conceptualized as a de-implementation initiative at the time it occurred. To enhance credibility of findings, participant perspectives were triangulated during analysis, and no substantial discrepancies were identified. Moreover, member-checking was incorporated to validate the accuracy, completeness, and alignment with participant experiences. Additionally, the process map was generated retrospectively at a single center, which may have resulted in omission of key components of the de-implementation process. Further, the Phase 2 expert panel did not include representation from low-income countries or Southeast Asia, which limited identification of context-specific considerations relevant to diagnostic de-implementation in these settings and reduced the framework’s generalizability. As a result, the framework may underrepresent process steps, determinants, and behavioral factors common to these settings. Prospective application of the framework across diverse geographic, cultural, and resource contexts will be necessary to identify these factors, support iterative refinement, and strengthen generalizability.

Lastly, future prospective application of the process map should examine factors contributing to long-term sustainability. The primary focus of this study was understanding the de-implementation process, and as a result, factors influencing long-term sustainment were not explored in depth. Once conventional cytogenetics was discontinued for routine ALL diagnosis, workflows, ordering practices, and infrastructure were reconfigured, making reintroduction of routine cytogenetics unlikely without substantial re-investment and workflow redesign. In addition, de-implementation was supported by institutional leadership and aligned with broader goals of improving resource utilization, diagnostic efficiency, and genomic classification, which likely facilitated sustainment of the change. Prospective application of the framework will be necessary to better understand determinants of long-term sustainability, adaptation, and maintenance across diverse settings.

In summary, advances in diagnostic technologies underscore the importance of considering de-implementation of traditional testing practices to improve care delivery and reduce low-value care. At a single center, de-implementation of conventional cytogenetics in the diagnosis of pediatric ALL following implementation of clinical genomics was feasible and achieved without adverse impact on patient outcomes. This experience informed development of a proposed process framework to guide future diagnostic de-implementation. Prospective application of this framework across diverse resource and cultural settings offers opportunities for iterative and contribution to the growing evidence base supporting de-implementation in cancer care. Ultimately, such efforts aim to enhance care delivery for children with cancer while improving health system efficiency.

## Data Availability

The datasets presented in this article are not publicly available because they contain information that could compromise the privacy and confidentiality of research participants. Requests for access to de-identified data may be considered on a reasonable request basis and subject to institutional and ethical approvals. Requests should be directed to Marta Salek (marta.salek@stjude.org).
