# Supplementary material for: Mapping the de-implementation of traditional diagnostic tests in pediatric acute lymphoblastic leukemia
Source: Front Oncol. 2026 Aug 17;16:1885970. doi: 10.3389/fonc.2026.1885970 (PMC13526624; doi:10.3389/fonc.2026.1885970)
Supplement: Supplementary file 1 [file Table1.docx]

**Supplemental File**

Mapping of the De-implementation of Traditional Diagnostic Tests in Pediatric Acute Lymphoblastic Leukemia

Marta Salek^1,2^*, Kenneth Busby^3^, Nathaniel Webb^4^, Dylan Graetz^1,2^, Charles G. Mullighan^5^, Thomas Alexander^4^, Nickhill Bhakta^1,2^, Megan C Roberts^6^+, Lu Wang^5^+

| **Table/Figure** | **Page** |
| --- | --- |
| Supplemental Table 1. Phase 1 semi-structured interview guide and corresponding CFIR (Consolidated Framework for Implementation Research) domains and constructs. | 2 |
| Supplemental Table 2. Phase 1 process map member-checking focus group questions. | 4 |
| Supplemental Table 3. Phase 2 focus group guide. | 5 |
| Supplemental Table 4. Phase 1 Interview Analysis Matrix/Summary Template by Participant and CFIR Construct. | 6 |
| Supplemental Table 5. External expert impressions of de-implementation. | 9 |

Supplemental Table 1. Phase 1 semi-structured interview guide and corresponding CFIR (Consolidated Framework for Implementation Research) domains and constructs.

| **Question** | | **Prompts** | **CFIR Domain/Construct** |
| --- | --- | --- | --- |
| 1 | Please think back to when cytogenetics were a routine component of the diagnostic work-up of pediatric ALL. How did the idea arise to discontinue routine cytogenetics at St. Jude? | a. Who initially proposed the idea? b. Do you remember when these conversations started? c. What did you initially think when you first heard the proposal?  - Did you support discontinuation? - What worries did you have? - What worries did others have?  d. How did the proposal gain momentum?  - What was the perceived benefit?  e. What evidence existed to help support discontinuing this process? f. What factors were considered by you and your colleagues?    - Was cost a concern?    - Were there any regulatory concerns?    - Did it impact participation in clinical trials? | Innovation (Domain)   - Innovation Evidence-Base (Construct) - Innovation Evidence-Base (Construct) - Relative Advantage (Construct)   Cross-cutting* |
| 2 | Do you remember who was involved in deciding to discontinue routine cytogenetics in pediatric ALL? | a. Who were the key individuals involved in making this decision?    - What role did St. Jude leadership play?    - What role did you play? b. Were your colleagues enthusiastic about discontinuing routine cytogenetics?    - Why were they enthusiastic? c. Were your colleagues hesitant about discontinuing routine cytogenetics?    - Why were they hesitant? d. Which departments were impacted by this process change? e. Were any experts external to St. Jude consulted?    - If so, what role did they play? f. What steps were taken to get everyone onboard with this decision? | Individuals (Domain)   - Leaders - Characteristics (Motivation) - Innovation Deliverer/Recipient   Outer Setting (Domain)   - Partnerships, Connections (Construct)   Inner Setting (Domain)   - Structural Characteristics (Construct)   Implementation Process (Domain)   - Engaging   Cross-cutting* |
| 3 | Can you please talk me through the process of de-implementation. How did this process impact your department or workflow? | a. What planning took place?    - Were there meetings or workshops that took place before routine cytogenetics were discontinued?    - How frequently were these sessions?    - Who was there?    - What was accomplished? b. Were any strategies used to facilitate the discontinuation of routine cytogenetics?  - Did professional roles change?   - Did anyone lose their job?   - Did anyone transition from one job to another?   - Did the infrastructure of your department change?   - Was anyone added to your team? - Was there any training involved?   - Were any materials or information developed about this initiative?  c. How long did the process take?    - Do you remember when it started? When it finished? d. Were there any obstacles or challenges faced during the process of discontinuation?    - Which team provided support to overcome these challenges? e. How was the process tracked? | Individuals (Domain)   - Innovation Deliverer/Recipient   Inner Setting (Domain)   - Structural Characteristics (Construct) - Access to Knowledge and Information (Construct) - Available Resources (Construct)   Implementation Process (Domain)   - Teaming (Construct) - Assessing Needs (Construct) - Assessing Context (Construct) - Planning (Construct) - Adaptability (Construct) - Reflecting & Evaluating (Construct)   Cross-cutting* |
| 4 | After routine cytogenetics was discontinued, what outcomes, if any, were tracked? | a. What impact did this have on patient care? b. What ensured this project was successful? c. How was this information fed back to the teams involved? | Individuals (Domain)   - Innovation Recipients (Construct)   Implementation Process (Domain)   - Reflecting & Evaluating (Construct)   Cross-cutting* |
| 5 | Looking back, how do you feel about the de-implementation process? | a. Is there anything you would have done differently? | Implementation Process (Domain)   - Reflecting & Evaluating (Construct)   Cross-cutting* |
| 6 | Do you think that routine cytogenetics could be discontinued in the diagnostic work-up of pediatric ALL at other centers, similar to what was done at St. Jude? | a. Have you shared the experience at St. Jude of discontinuing routine cytogenetics with colleagues at other centers?    - What has prevented other centers to discontinue routine cytogenetics in pediatric ALL?    - What barriers do these centers face?  - When you speak with colleagues at other centers, what do you share about this experience? | Outer Setting (Domain)   - Partnerships, Connections (Construct)   Cross-cutting* |
| 7 | Is there anything additional you would like to share about this topic? |  | Cross-cutting* |
|  | *Legend: CFIR – Consolidated Framework for Implementation Research. Cross-cutting refers to constructs that may apply across CFIR domains, informed by the Integrated Behavioral Model, exploring constructs such as personal agency, attitudes, and norms.* | | |

Supplemental Table 2. Phase 1 process map member-checking focus group questions.

| **Question** | | **Prompts** |
| --- | --- | --- |
| 1 | Does the map accurately capture the process of de-implementation of routine cytogenetics at St. Jude? |  |
| 2 | Is there anything missing from the process map that should be included? |  |
| 3 | Do you think an expert from an outside institution could easily understand the process of de-implementing routine cytogenetics based on this map? | a. What could improve communication? |
| 4 | Is there anything that would be removed from this process map that you feel is unnecessary? |  |
| 5 | Is there anything additional you would like to share? |  |

Supplemental Table 3. Phase 2 focus group guide.

| **Question** | | **Prompts** | **CFIR Domain/Construct** |
| --- | --- | --- | --- |
| 1 | What are your impressions about the de-implementation of routine cytogenetics in pediatric ALL? | a. How does discontinuing routine cytogenetics in pediatric ALL resonate with you?    - Has your center or other centers done this? | Cross-Cutting*  Outer Setting (Domain)   - Partnerships, Connections (Construct) |
| 2 | Based on the information presented, do you think that routine cytogenetics could be discontinued at your center? | a. Do you think routine cytogenetics for pediatric ALL should be discontinued at your center? b. What barriers do you anticipate? c. What would contribute to successful discontinuation of routine cytogenetics? d. Is there anything that is confusing? e. Is there anything that needs clarification? f. Are there any components you think are missing? | Cross-Cutting*  Innovation (Domain)   - Relative Advantage (Construct) - Complexity (Construct) - Design (Construct)   Implementation Process (Domain)   - Assessing Context (Construct) |
| 3 | Can you think of a diagnostic test that could or should be de-implemented at your center in childhood cancer? | a. How does this process map related to de-implementing a diagnostic intervention in childhood cancer resonate with you? b. Does it align with how the process would look like at your site? c. What barriers do you anticipate? d. What would contribute to successful discontinuation of routine cytogenetics? | Innovation (Domain)   - Relative Advantage (Construct) - Design (Construct)   Inner Setting (Domain)   - Compatibility (Construct)   Implementation Process (Domain)   - Assessing Context, Planning (Construct) |
| 4 | Do you have any questions about the process map that was presented? | a. Is there anything that is confusing? b. Is there anything that needs clarification? c. Are there any components you think are missing? | Cross-Cutting*  Innovation (Domain)   - Design (Construct) - Complexity (Construct) |
| 5 | Is there anything additional you like to share about this topic? |  | Cross-Cutting* |
|  | *Legend: CFIR – Consolidated Framework for Implementation Research. Cross-cutting refers to constructs that may apply across CFIR domains, informed by the Integrated Behavioral Model, exploring constructs such as personal agency, attitudes, and norms.* | | |

Supplemental Table 4. Phase 1 Interview Analysis Matrix/Summary Template by Participant and CFIR Construct.

| **Interview Question** | **Question Prompt** | **CFIR Domain** | **CFIR Construct** | **Interview #1** | **Interview #2** | **Interview #X…** |
| --- | --- | --- | --- | --- | --- | --- |
| 1 | How did the idea arise to discontinue routine cytogenetics at St. Jude? | Innovation | Innovation Source |  |  |  |
| 1a | Who initially proposed the idea? | Innovation | Innovation Source |  |  |  |
| 1b | Do you remember when those conversations started? | N/A | N/A |  |  |  |
| 1c | Corss- think when you first heard the proposal? | Cross-Cutting | |  |  |  |
| 1d | How did the proposal gain momentum? | N/A | N/A |  |  |  |
| 1di | What was the perceived benefit? | Innovation | Relative Advantage |  |  |  |
| 1e | What evidence existed to help support discontinuing this process? | Innovation | Evidence-Base |  |  |  |
| 1f | What factors were considered by you and your colleagues? | Cross-Cutting | |  |  |  |
| 2 | Do you remember who was involved in deciding to discontinue routine cytogenetics in pediatric ALL? | Individuals | Leaders |  |  |  |
| 2a | Who were they key individuals involved in making this decision? | Individuals | Leaders |  |  |  |
| 2b | Were your colleagues enthusiastic about discontinuing routine cytogenetics? | Individuals | Characteristic (Motivation) |  |  |  |
| 2c | Were your colleagues hesitant about discontinuing routine cytogenetics? | Individuals | Characteristic (Motivation) |  |  |  |
| 2d | Which departments were impacted by this process change? | Individuals | Innovation Deliverer/Recipient |  |  |  |
| 2e | Were there any experts external to St. Jude consulted? | Outer Setting | Partnerships, Connections |  |  |  |
| 2ei | If so, what role did they play? | Outer Setting | Partnerships, Connections |  |  |  |
| 2f | What steps were taken to get everyone onboard with this decision? | Implementation Process | Engaging |  |  |  |
| 3 | Can you please talk me through the process of de-implementation. How did this process impact your department or workflow? | Inner Setting | Structural Characteristics |  |  |  |
| 3a | What planning took place? | Implementation Process | Teaching, Assessing Needs, Assessing Context, Planning |  |  |  |
| 3ai | Were there meetings or workshops that took place before routine cytogenetics were discontinued? |  |  |  |  |  |
| 3b | Were there strategies used to facilitate the discontinuation of routine cytogenetics? | Implementation Process | Teaming |  |  |  |
| 3bi | Did professional roles change? | Individuals | Innovation Deliverer/Recipient |  |  |  |
| 3bii | Did the infrastructure of your department change? | Inner Setting | Structural Characteristics |  |  |  |
| 3biii | Was there any training involved? | Inner Setting | Access to Knowledge and Information |  |  |  |
| 3biv | Were there any materials or information developed about this initiative? | Inner Setting | Available Resources |  |  |  |
| 3c | How long did this process take? | N/A | N/A |  |  |  |
| 3d | Were there any obstacles or challenges faced during the process of discontinuation? | Cross-Cutting | |  |  |  |
| 4 | After routine cytogenetics was discontinued, what outcomes, if any, were tracked? | Innovation Process | Reflecting & Evaluating |  |  |  |
| 4a | What impact did this have in patient care? | Individuals | Innovation Recipient |  |  |  |
| 4b | What ensured this project was successful? | Cross-Cutting | |  |  |  |
| 4c | How was this information fed back to the teams involved? | Innovation Process | Reflecting & Evaluating |  |  |  |
| 5 | Looking back, how do you feel about the de-implementation process? | Innovation Process | Reflecting & Evaluating |  |  |  |
| 5a | Is there anything you would have done differently? | Cross-Cutting | |  |  |  |
| 6 | Do you think that routine cytogenetics could be discontinued in the diagnostic work-up of pediatric ALL at other centers, similar to what was done at St. Jude? | Cross-Cutting | |  |  |  |
| 6a | Have you shared the experience at St. Jude of discontinuing routine cytogenetics with colleagues at other centers? | Outer Setting | Partnerships, Connections |  |  |  |
| 6ai | What has prevented other centers from discontinuing routine cytogenetics in pediatric ALL? | Cross-Cutting | |  |  |  |
| 6b | When you speak with colleagues at other centers, what do you share about this experience? | Outer Setting | Partnerships, Connections |  |  |  |
| 7 | Is there anything additional you would like to share about this topic? | Cross-Cutting | |  |  |  |
|  | *Legend: CFIR – Consolidated Framework for Implementation Research. Cross-cutting refers to constructs that may apply across CFIR domains, informed by the Integrated Behavioral Model, exploring constructs such as personal agency, attitudes, and norms.* | | | | | |

Supplemental Table 5. Phase 2 external expert impressions of the generated process maps and perceptions of de-implementation.

| **Theme/Subtheme** | **Quotation** |
| --- | --- |
| General Impressions of process maps | I think [the process maps were] done really nicely. Step-by-step, the way you explained it, instead of just saying one day we're not going to do it. I think that's the biggest lesson here, as each institution is trying to figure out whether they should take away [karyotyping], instead of a couple of people making that decision and just letting the rest of the team know, this process that you've explained and the way you all did it there was very impressive. [The process] should take everybody's input into consideration. (Phase 2 Participant 8, HIC) |
|  | It's very interesting to see all these experiences because I haven't de-implemented a test yet, but [will] think of that for the future. (Phase 2 Participant 13, HIC) |
| Potential Opportunities for De-Implementation | For me none currently, because we have the bare minimum. (Phase 2 Participant 1, LMIC) |
|  | Well, what we [stopped] doing was measuring galactomannan to diagnose Aspergillus because it was a costly test. The information that it gave was not according to, to the literature [… the infectious disease specialists] decided with the lab that this test will no longer be done. But we did not follow a process like this […] instead, we substituted [the galactomannan] test for other tests. (Phase 2 Participant 12, UMIC) |
| Potential applicability of process maps to low-resourced settings | I think it's a well thought [out] process. And I think it's something that we can also benefit from on this part of the world. (Phase 2 Participant 1, LMIC) |
|  | So de-implementation is very interesting for us because due to our protocols, we should the cytogenetics abroad and it costs a lot of money. (Phase 2 Participant 3, LMIC) |
|  | [With] cytogenetics we have almost 40% failure […] I think that [at] some point we [need to stop] doing [cytogenetics … and] replace [it] for another technique. We need to optimize resources […find] the optimal test, the best way [of] doing it. (Phase 2 Participant 14, HIC) |
| *Abbreviations: LMIC – lower-middle-income country; HIC – high-income country; UMIC – upper-middle-income country.* | |
